# White blood cell image analysis for infection detection based on virtual hexagonal trellis (VHT) by using deep learning

**DOI:** 10.1038/s41598-023-44352-8

**Published:** 2023-10-19

**Authors:** Shahid Rashid, Mudassar Raza, Muhammad Sharif, Faisal Azam, Seifedine Kadry, Jungeun Kim

**Affiliations:** 1 Department of Computer Science, COMSATS University Islamabad, Wah Campus, Islamabad, 47040 Pakistan; 2 Department of Applied Data Science, Noroff University College, Kristiansand, Norway; 3 Department of Electrical and Computer Engineering, Lebanese American University, Byblos, Lebanon; 4 Artificial Intelligence Research Center (AIRC), College of Engineering and Information Technology, Ajman University, Ajman, United Arab Emirates; 5 Department of Software, Kongju National University, Cheonan, 31080 Korea

**Keywords:** Computational science, Computer science

## Abstract

White blood cells (WBCs) are an indispensable constituent of the immune system. Efficient and accurate categorization of WBC is a critical task for disease diagnosis by medical experts. This categorization helps in the correct identification of medical problems. In this research work, WBC classes are categorized with the help of a transform learning model in combination with our proposed virtual hexagonal trellis (VHT) structure feature extraction method. The VHT feature extractor is a kernel-based filter model designed over a square lattice. In the first step, Graft Net CNN model is used to extract features of augmented data set images. Later, the VHT base feature extractor extracts useful features. The CNN-extracted features are passed to ant colony optimization (ACO) module for optimal features acquisition. Extracted features from the VHT base filter and ACO are serially merged to create a single feature vector. The merged features are passed to the support vector machine (SVM) variants for optimal classification. Our strategy yields 99.9% accuracy, which outperforms other existing methods.

## Introduction

Leukocytes or WBC are the vital constituent of human immune system. WBC help identify possible infections in humans. Hematologists^1^ use WBC images for syndrome analysis in the therapeutic domain. Accurate analysis of WBCs plays a key role in estimating diseases in terms of class and stage. Emerging artificial intelligence (AI)-based techniques are assisting in the counting and analysis of WBC cell structures. Hexagonal image processing models^2,3^ for WBC image classification are an innovative field of study. Image interpolation techniques transform square image structures into a hexagonal format. In this transformation, alternative rows of image pixels are shifted by half the distance. This transformation^4,5^ requires additional space and computational power to handle sampling data^6^. Existing classification techniques lack the utilization of hexagonal trellis-based structures for WBCs^7,8^. Moreover, these classical techniques are affected by imbalanced illumination^9,10^ and low contrast with high background interfaces^11,12^. The hexagonal structure retains an inherent capability of extracting comparatively useful information compared to the square structure.

**Figure 1:**
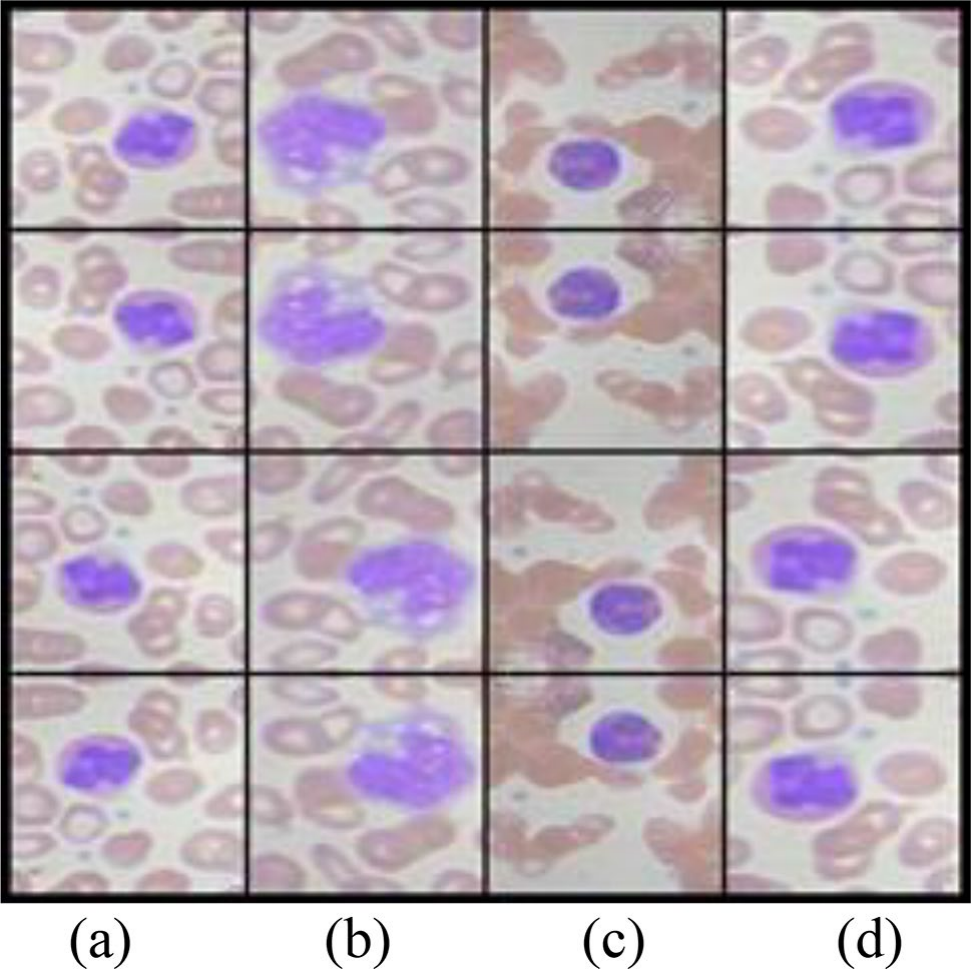
WBC four classes (**a**) Eosinophil (**b**) Lymphocyte (**c**) Monocyte (**d**) Neutrophil.

The conventional examination of WBC count or infection analysis is typically performed through manual inspection^13^ of smear images in the histopathology lab. WBCs smear images are collected using an L2 microscope. The ALL-IBD-2 dataset^14^ presents four classes of WBC images, as shown in Fig. 1. These images exhibit different orientations with respect to the background's black shade level. Low contrast and imbalanced illumination are noticeable within the cytoplasm of WBCs. Each class in Fig. 1 is accompanied by a set of four sample images. These WBC images depict irregularly shaped nuclei with a blue contrast. The presence of infection within the WBC nucleus reflects the cause and severity of the disease during analysis. Shape deformation of the WBC nucleus due to infection serves as the primary indicator for disease identification. Given the large volume of WBC observations and the low-quality of the images, the manual inspection process can sometimes result in incorrect diagnoses. Consequences of incorrect diagnoses include prescribing the wrong medication, misinterpreting the disease's origin, and inaccurately counting WBCs. Such errors can pose risks to the patient's life.

To overcome the issues mentioned in the preceding para, this research introduces a framework to provide an ease in the analysis and diagnosis of infection inside the WBCs nucleus. Our framework also handles irregular illumination effects generated during image acquisition process^15^ for accurate classification. In this work, a hexagonal structured filter is presented to extract feature in combination with a pre trained CNN model GraftNet. The VHT feature extractor is primarily based on x–y gradient orientations effect. The extracted features from Graft Net^16^ and VHT extractor are serially fused together. The fused subspace batches of features are feed to the multi layered classifier for WBC nucleus analysis and classification. The contribution of our work is as under:

- A new feature extractor based on virtual hexagonal trellis (VHT-FE) is proposed as a hand-crafted feature extractor.
- The data augmentation is accomplished to upsurge the images in each class of dataset
- Features are extracted from the suggested VHT base feature extractor and GraftNet.
- Optimized features are collected using ACS feature selection approach to analyze the proposed model for WBC classification.

The organization of the manuscript is: The section "Introduction" offers a transitory overview to the domain and sketches the selected approach. Section "Related work" reviews recent feats in associated fields. This section highlights existing machine learning means for feature mining. In the third section, the test results are presented using a dataset of 10,000 blood smear pictures, with 2500 images in each of the four classes. Section "Results and discussion" critically analyzes and compares the results with current methods. The conclusion section debates the obtained results and future directions.

## Related work

The visual quality of WBC smear images plays a vital rule in accurate and precise histopathological diagnosis. Poor image quality obtained from L2-microscopes can negatively influence the accuracy of WBC smear image analysis^17^. During collection, microscopic smear images of white blood cells (WBCs) can sometimes appear dull^18^. Feature extraction is an imperative step in the field of digital image processing before selection of features and classification^19,20^.

The literature review encompasses a wide range of approaches, including pre-processing, morphological operations, attribute analysis, and feature extraction, for traditional WBC classification^21^. So far, few efforts are found that use deep learning techniques for white blood nucleus recognition from the given blood smear images. Some authors have evaluated various convolutional neural networks (CNNs), such as LeNet5, AlexNet, and GoogLeNet, for WBC classification. These networks have the disadvantage of high computational cost^22^. The empirical findings in this research contribute to an improved understanding of features acquisition and make notable contributions to automated WBC prediction. In current hematology, analyzers such as Siemens ADVIA 2120i and Sysmex XE-series^23^ are commonly used. However, these analyzers often suffer from poor resolution and are limited to certain classes of leukocyte types. Continuous updates of these systems rely on expensive chemicals processes^24^. Yampri et al.^25^ have focused on feature extraction using a combination of eigenvalues and parametric functions. Falcon et al.^26^ presented their work on extracting the shape of the nucleus in WBCs based on contours and regions. Some studies have focused on the boundary of the WBC nuclei, while others have considered the segmention part of the nuclei. Multilayered classifiers including SVM and KNN were used to achieved accuracy more than 96%. Habibzadeh et al.^27^ dig up nucleus from low-resolution images, applied three set of feature spaces functions, and employed SVM as a classification. The highest classification rate was obtained from the DT-CWT feature set, combined with SVM, causing in 84% classification rate. Su et al.^28^ operated on texture elements based on geometric features, color intensity, and local direction patterns (LDP) extracted from segmented cells. These features were handled by CNN, resulting in an overall accuracy of 99.11%. Gautam et al.^29^ applied simple shape-based features of segmented WBC nuclei from split images and used a categorization principle for WBC type classification. Prinyakupt et al.^30^ executed the Greedy Search Algorithm (GSA) for the extraction of different features, combining cytoplasm and nucleus information from WBCs. Ravikumar et al.^31^ predicted WBCs based on the nuclei, engaging relevance vector machine (RVM). Ravikumar^32^ conducted a rapid and enhanced study by utilizing the Fast-RVM to identify WBCs from the main cell. Zheng et al.^33^ presented the FHF method for localized feature extraction from medical images. Liu and Yang^34^ proposed the FCB model for saliency object feature detection and extraction from WBC smear images. Supervised learning methods mainly contribute to the field through the use of SVM^20^. Zhen et al.^35^ contributed to improving boundary segmentation accuracy by employing the median color method and detecting low-intensity edges to handle vague boundaries. Bau et al.^36^ trained an SVM model on a randomly selected set of 200 images for classification trees.

Recent approaches have focused on deep CNN models, which have proven to be more skillful at extracting information from images. High-gradient-based data^24,37^ has made significant contribution to image classification. The implications of these methods extend to image classification^38,39^, object detection^40^, boundary extraction^41^, edge detection^42^, and image segmentation^43^.

Other approaches include transformer-based methods^44^, model-based approaches^45^, graph-based approaches^46^, learning-based approaches^47^, and entropy-based approaches^34^. Additionally, fully convolutional networks (FCN)^48^ have been used for image-to-pixel-level classification to reduce the computational workload of pre-processing. Guerrero-Pena et al.^49^ presented a weighted map for a weighted cross-entropy loss function used for imbalanced parametric correction, while Chen et al.^50^ proposed a contour-aware FCN for the segmentation of medical part images.

Hexagonal patterns can be perceived in numerous natural scenarios such as the hexagonal structure of honeycombs produced by honey bees. The hexagon embraces noteworthy significance in various fields. Hexagonal grids are widely utilized in the graph theory, spatial modeling, and computer algorithms. The aesthetic appeal of hexagonal grids makes them popular in computer graphics, visualization, and image processing. In the context of medical image analysis, the hexagonal trellis has grown reputation due to its natural occurrence, structural befits, and various technological applications. The hexagonal trellis offers not only practical benefits but also visual appeal, making it a suitable choice in medical image analysis. The motivation behind incorporating hexagonal patterns lies in their natural occurrence, structural advantages, industrial and technological applications, and aesthetic appeal. These factors contribute to their utilization in medical image analysis, including the exact application we are focusing in our research.

The categorization of WBC images postures noteworthy challenges, mainly in the precise acquisition of other WBC subparts for example RBCs, cytoplasm, and platelets. The significant objectives in leukocyte image analysis is to attain accurate classification. Current means often meet limitations especially when dealing with a larger dataset. These approaches can result in information loss in the transformation of images between square and hexagonal lattices. Accuracies are classically assessed in relation to efficiency, considering the number of leukocyte images. One key area where current methods often suffer is feature extraction, mainly in the diagonal direction, owing to the square image trellis. The primary motivation of our research is to address the limitations such as improve leukocyte classification accuracy, and lessen information loss during image transformation. By enhancing feature extraction, including diagonal direction analysis, we aim to develop an effective approach for WBC image prediction.

In conclusion, all the techniques presented in the preceding section may lead to inefficient image analysis. The proposed method aims to reduce the computational cost and preserve information by employing a hexagonal feature extraction and classification approach in the upcoming sections.

## Proposed method

**Figure 2:**
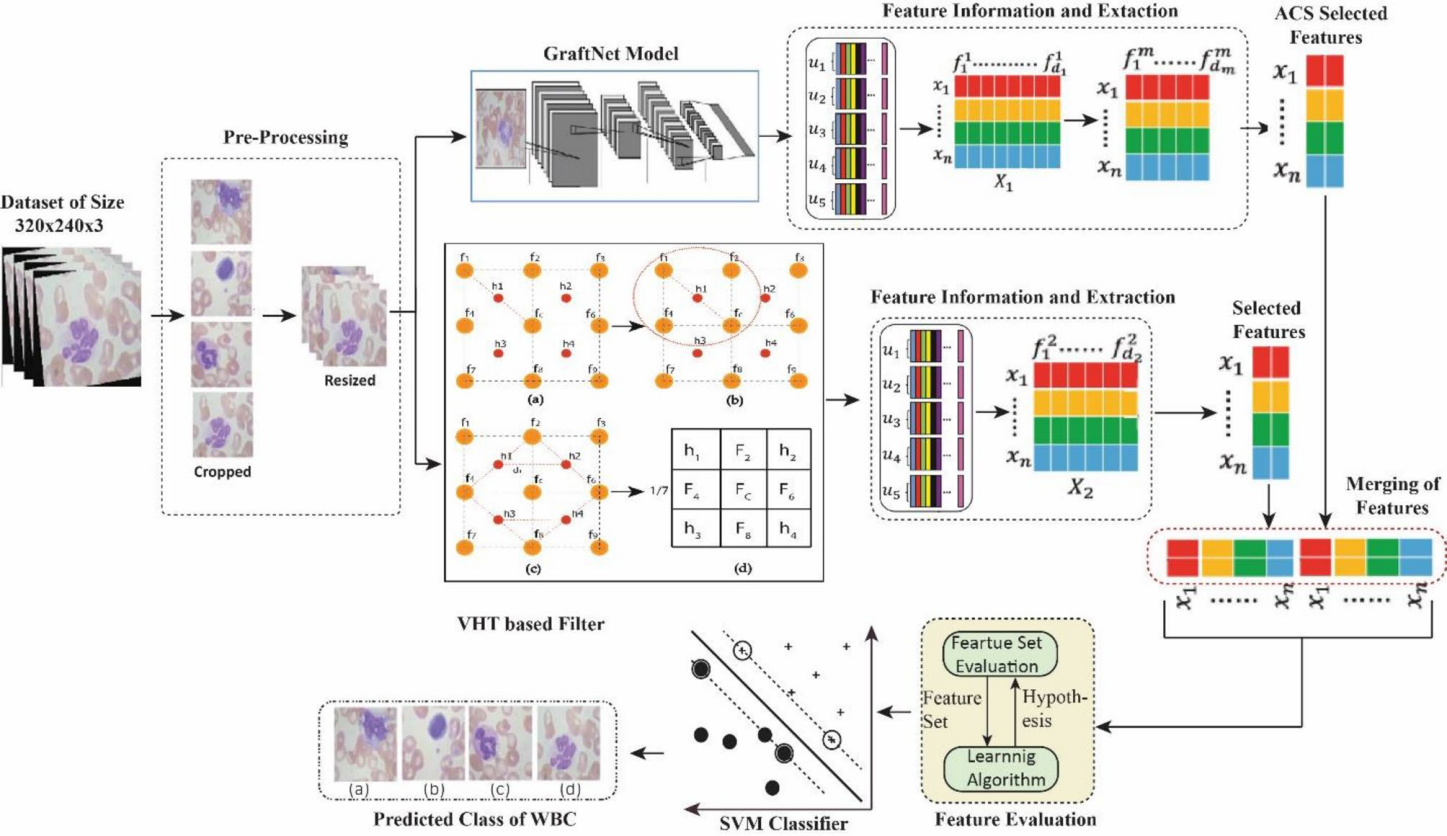
Proposed model based on virtual hexagonal trellis (VHT) in combination with Graftnet pretrained model.

The proposed model VHT-FE is presented with GraftNet CNN model for feature extraction. This hybrid approach is used for analysis of infection present inside the nucleus of WBC. The WBC dataset and the model configuration are presented in this section. The block diagram of the anticipated model is presented in Fig. 2. This model is based on the steps including Pre-processing (cropping, resizing and augmentation), feature extraction (both CNN-based and VHT based), optimized feature selection using ACO and classification.

### Pre-processing

**Figure 3:**
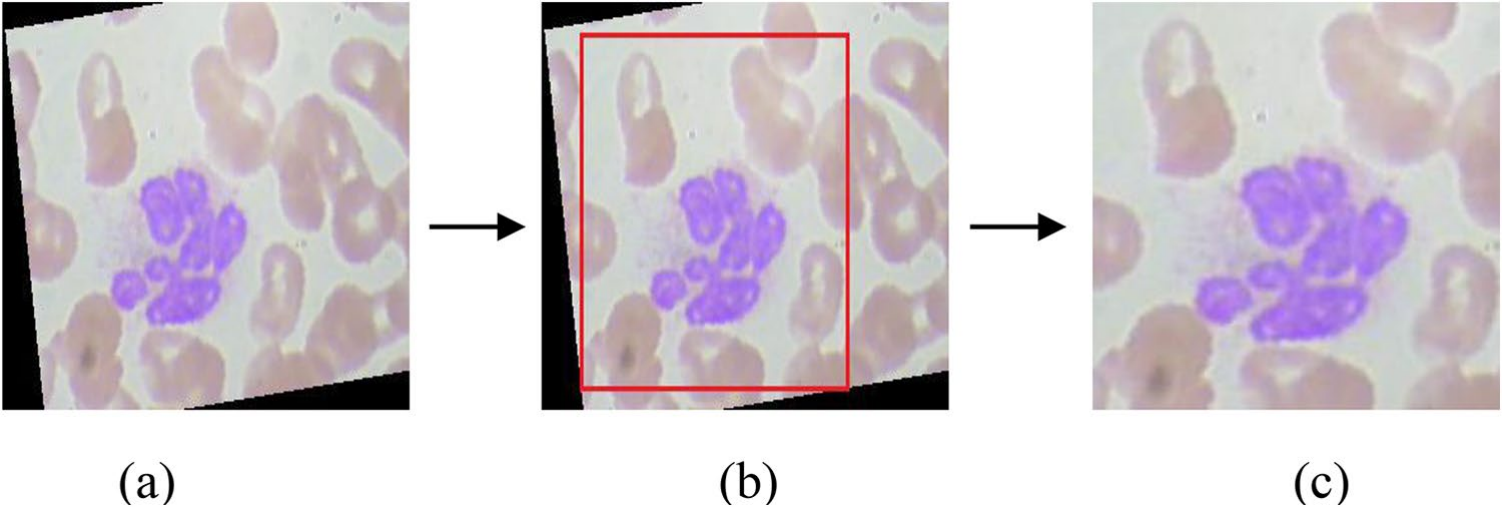
Pre-processing over ALL-IBD2 Dataset (**a**) Original Image (**b**) Manual cropping (**c**) Annotated Image.

WBC images contain irregular lighting effects due to imbalanced dark areas, as shown in Fig. 3a, acquired from an L2 microscope. The complete WBC dataset, named ALL_IBD2, is publicly available. It consists of four classes including (a) Neutrophil, (b) Eosinophil, (c) Lymphocyte, and (d) Monocyte. All class contain around 600–650 images each. To normalize these WBC images, a cropping function is used to remove the extra dark regions from the background, as depicted in Fig. 3b. This dataset is augmented to 2500 images per class, resulting in a total of 10,000 images. The annotated WBC images in Fig. 3c have a size of 200 × 200 pixels.

### Feature extraction

The augmented WBC dataset is supplied to the WHT-FE and a pretrained GraftNet model separately. The features mined by the GraftNet are then passed to ACO algorithm for optimal feature selection. The features selected by ACO are fused with the VHT-FE features in a sequential manner. The analysis and experimental evaluation of these fused features for classification are discussed in the upcoming subsections. This policy for feature selection increases the overall classification accuracy.

#### Pre-trained L2-graft net

**Figure 4:**
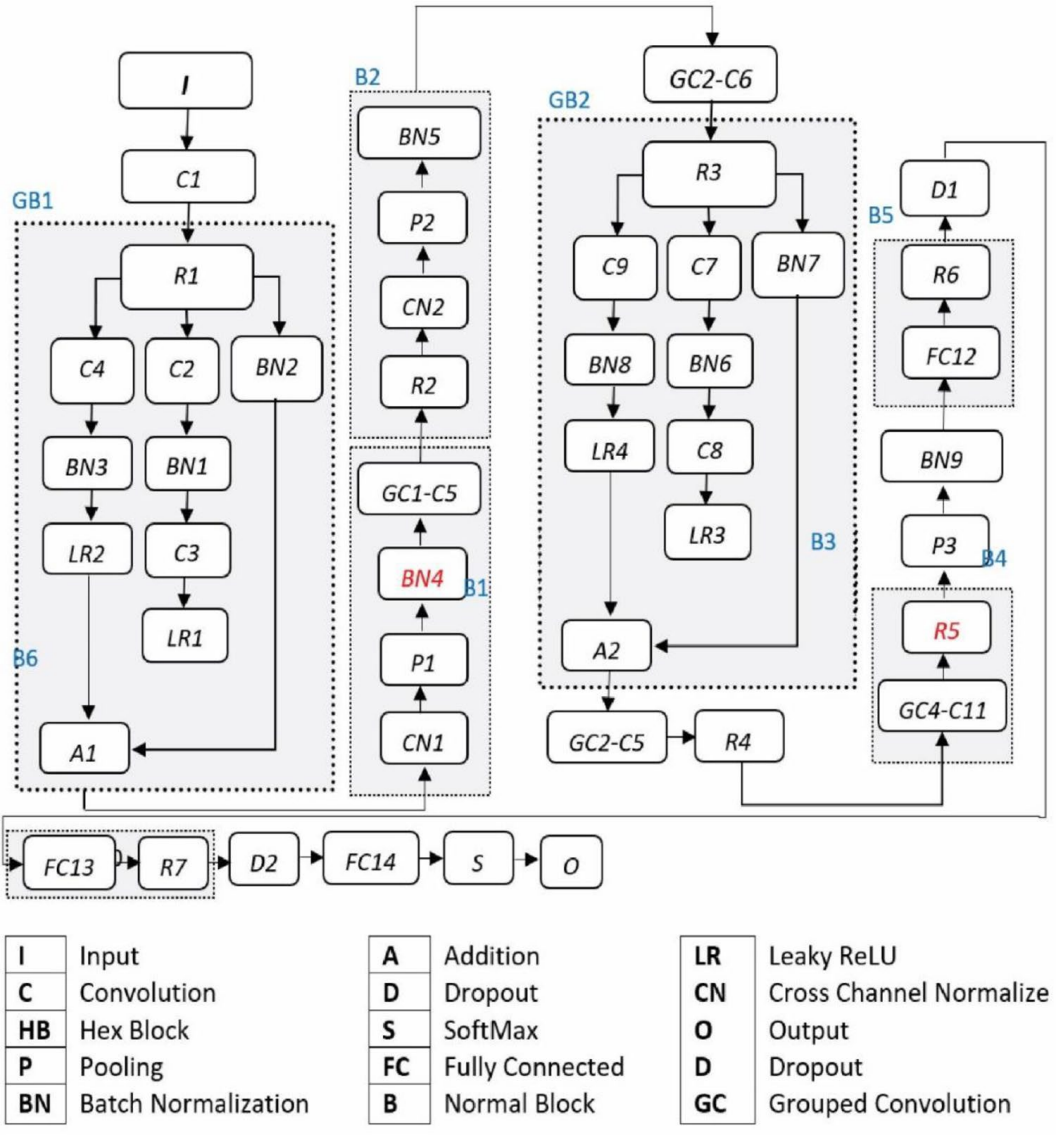
GraftNet architectural visualization.

Graft Net is modified form of two famous CNN based architectures such as Alex net^51^ and some layer contribution of Squeezed Net^52^. The architecture of the pretrained model AlexNet consists of three pooling layers at different levels, seven ReLU layers, five convolutional layers, two dropout layers, a single SoftMax layer, and three fully connected layers in the entire model. In the Graft Net architecture, some batch normalization layers are added from a fire module-based structure to further refine it, as shown in Fig. 4.

#### Proposed VHT feature extractor

The proposed virtual hexagonal trellis (VHT) for feature extraction is a comprehensive approach. Traditional approaches, such as square and hexagonal methods, are only capable of producing rectangular visuals. Currently, there is no existing method for defining a hexagonal structure for dealing with medical images. In the proposed work, a hexagonal trellis is introduced to overlay the inherent square grid structure of the image. The stable type of hexagonal trellis is inspired by the structure of bee colonies^53^. Additionally, this study takes into consideration the color properties, such as intensity values, contrast (gradation of energy), and correlation between textures in WBC images.

**Figure 5:**
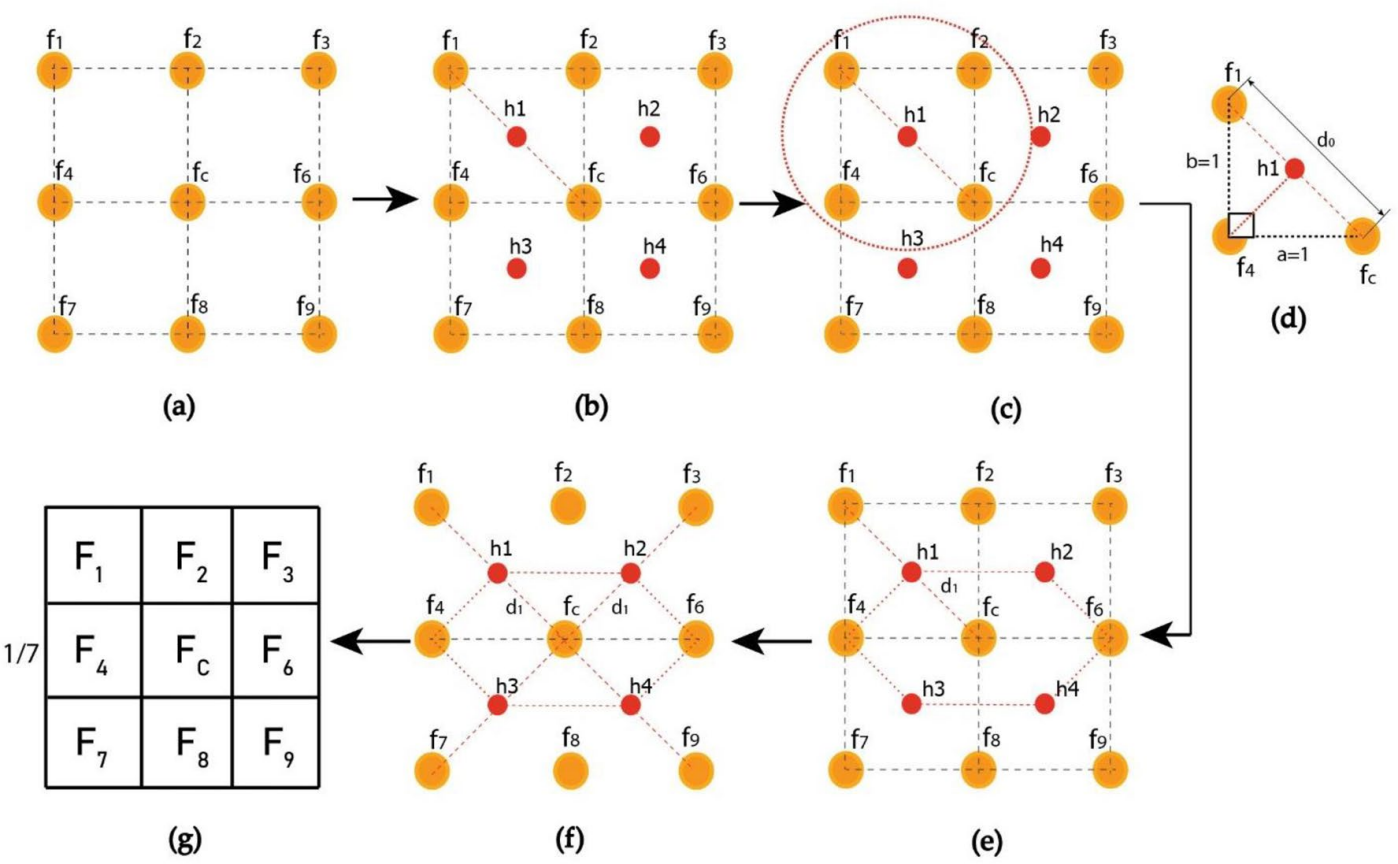
Convolution operator for feature extraction (COFE) over square grid Structure.

The present work underlines the optimal performance of the proposed feature extractor. The VHT is implemented using a convolution operator to transform an image with a rectangular shape. Figure 5 illustrates the evolution of the VHT structure over the square grid. The fundamental operational structure for all image-capturing devices is based on a square grid. The steps employed for formulating the VHT base filter/mask are briefly described below.

##### Generating virtual elements (VE)

In this stage, a sample size of 3 × 3 square grid form the image pixels is utilized. These virtual pixels and elements are positioned at equidistant between the actual square-shaped pixels, as seen in Fig. 5a. An actual square grid without any intensity values is shown in Fig. 5b, along with the virtual pixels.

##### Assigning intensity to VE

Following the previous stage, the newly generated pixels depicted in Fig. 5c and d represent the virtual pixels or elements with average central intensities. The intensity value of these virtual pixels is considered as the mean intensity of central pixel and its neighboring pixels.

##### Convolutional operator for feature extraction (COFE)

The assigned intensities to the newly generated virtual elements are utilized in the construction of the feature extractor in the proposed technique. A virtual hexagonal trellis is superimposed on the original square structure, as illustrated in Fig. 5e. The feature extractor operator, depicted in Fig. 5f, is generated from this hexagonal trellis. It convolutes over the image and accumulates textural features based on intensity, energy, and correlation. This hexagonal trellis-based architecture replaces the square grid, allowing for various mask formulations, as shown in Fig. 5g.

##### Sample square structure (SSS)

**Figure 6:**
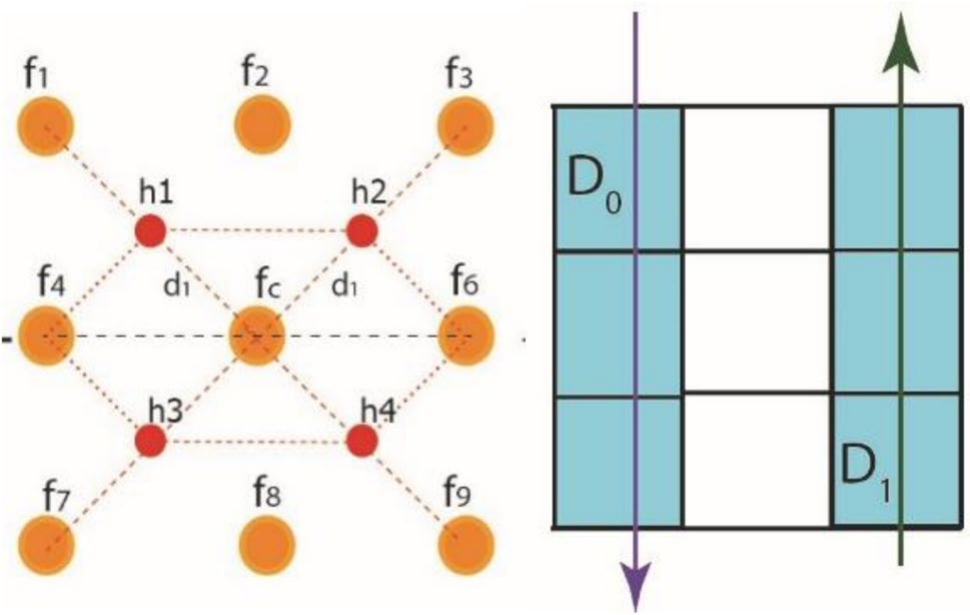
Gradient effect of real and virtual pixels along y-axis.

For the feature extractor/operator, this work employs virtual pixels over a sample square lattice of an input image. Figure 6 shows these virtual pixels in hexagonal patterns to calculate the gradient effect of pixel intensities in vertical direction.

1 $$\nabla M=\left[\begin{array}{c}\frac{\partial f}{\partial x}\\ \frac{\partial f}{\partial y}\end{array}\right]$$

2 $$\frac{\partial f}{\partial x}={M}_{x}= Variable\, along\, x-axis$$

3 $${M}_{x}=\left[{(f}_{6}+{h}_{2}+{h}_{4})-{(f}_{4}+{h}_{1}+{h}_{3})\right]$$

**Figure 7:**
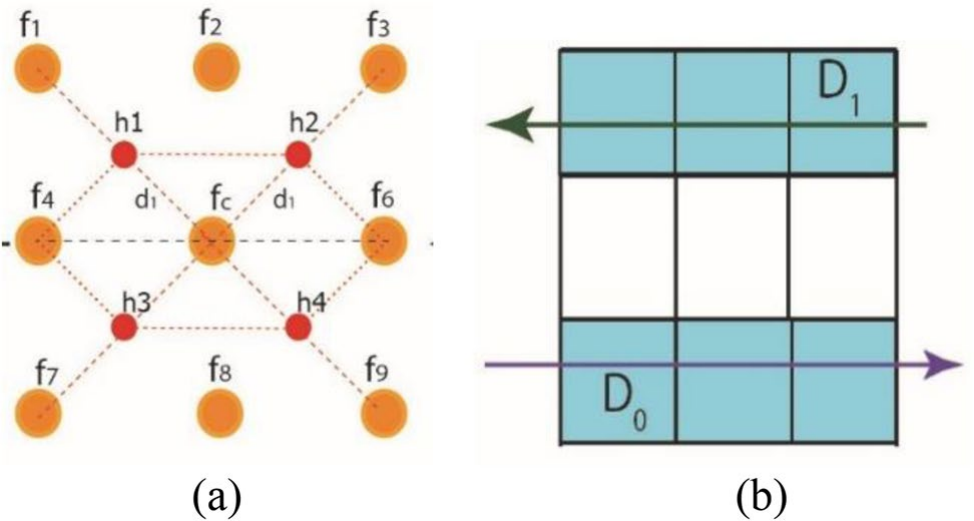
Gradient effect of real and virtual pixels (**a**) Hexagonal grid with virtual pixels (**b**) Intensity calculation along x- axis.

The average intensity value of the real pixel and its corresponding neighboring pixel in hexagonal grid structure is presented in Fig. 7a as VEV. Figure 7b shows gradient effect of newly generated pixels over a virtual hexagonal structure along the horizontal axis.

4 $$\frac{\partial f}{\partial y}={M}_{y}= Variable\, along\, y-axis$$

5 $${M}_{y}=\left[{h}_{3}+{h}_{4})-({h}_{1}+{h}_{2})\right]$$

In Fig. 5f, all values of real element of the square grid are substituted with values of the virtual element variables (VEV) ${"h}_{n}$" of ${"f}_{n}"$. Similarly, using real elements from the square gird, all VEV are updated, and Eqs. (3) and (5) are reformed by substituting the value of virtual pixel intensities from Eqs. (6) to (9) as follows:

6 $${h}_{1}= \frac{{f}_{1}+{f}_{c}}{2}$$

7 $${h}_{2}= \frac{{f}_{3}+{f}_{c}}{2}$$

8 $${h}_{3}= \frac{{f}_{7}+{f}_{c}}{2}$$

9 $${h}_{4}= \frac{{f}_{9}+{f}_{c}}{2}$$

We obtain the mathematical derivation of "x" and "y" by entering all values of VEV in Eqs. (3) and (5).

10 $${M}_{x}=\left[{(f}_{6}+\frac{{f}_{3}+{f}_{c}}{2}+\frac{{f}_{9}+{f}_{c}}{2})-{(f}_{4}+\frac{{f}_{1}+{f}_{c}}{2}+\frac{{f}_{7}+{f}_{c}}{2})\right]$$

11 $${M}_{x}=\frac{1}{2}\left[{(2f}_{6}+{f}_{3}+{f}_{c}+{f}_{9}+{f}_{c})-{(2f}_{4}+{f}_{1}+{f}_{c}+{f}_{7}+{f}_{c})\right]$$

12 $${M}_{x}=\frac{1}{2}\left[{(2f}_{6}+{f}_{3}+{f}_{c}+{f}_{9}+{f}_{c}-{2f}_{4}-{f}_{1}-{f}_{c}-{f}_{7}-{f}_{c})\right]$$

13 $${M}_{x}=\frac{1}{2}\left[{(2f}_{6}+{f}_{3}+2{f}_{c}+{f}_{9}-{2f}_{4}-{f}_{1}-2{f}_{c}-{f}_{7})\right]$$

14 $${M}_{x}=\frac{1}{2}\left[{(2f}_{6}+{f}_{3}+{f}_{9}-{2f}_{4}-{f}_{1}-{f}_{7})\right]$$

15 $${M}_{x}=\frac{1}{2}\left[\begin{array}{ccc}-1& 0& 1\\ -2& 0& 2\\ -1& 0& 1\end{array}\right]$$

Similarly, values of VEV are replaced in Eq. (5) in the same manner to get gradient effect of real and virtual elements along y-axes. Replacing the values of VEV from Eqs. (6)–(9) in Eq. (5),

16 $${M}_{y}=\left[\frac{{f}_{7}+{f}_{c}}{2}+\frac{{f}_{9}+{f}_{c}}{2})-(\frac{{f}_{1}+{f}_{c}}{2}+\frac{{f}_{3}+{f}_{c}}{2})\right]$$

17 $${M}_{y}=\frac{1}{2}\left[{f}_{7}+{f}_{c}+{f}_{9}+{f}_{c})-({f}_{1}+{f}_{c}+{f}_{3}+{f}_{c})\right]$$

18 $${M}_{y}=\frac{1}{2}\left[{f}_{7}+{2f}_{c}+{f}_{9})-({f}_{1}+{2f}_{c}+{f}_{3})\right]$$

19 $${M}_{y}=\frac{1}{2}\left[{f}_{7}+{2f}_{c}+{f}_{9}-{f}_{1}-{2f}_{c}-{f}_{3})\right]$$

Cancellation of ${f}_{c}$ took place, due to inverse effect of gradient directions.

20 $${M}_{y}=\frac{1}{2}\left[{f}_{7}+{f}_{9}-{f}_{1}-{f}_{3})\right]$$

21 $${M}_{y}=\frac{1}{2}\left[\begin{array}{ccc}1& 2& 1\\ 0& 0& 0\\ -1& -2& -1\end{array}\right]$$

The cumulative gradient effect is demonstrated by Eqs. (15) and (20) by employing VEV in the 'x' and 'y' directions, respectively. Similarly, Eqs. (15) and (21) generate aggregate effect of the filter along x-axis and y-axis as a kernel. Equation (24) also demonstrates the combined effect of the two kernels.

22 $${M}_{xy}=\frac{1}{2}\left[\begin{array}{ccc}-1& 0& 1\\ -2& 0& 2\\ -1& 0& 1\end{array}\right]+\frac{1}{2}\left[\begin{array}{ccc}1& 2& 1\\ 0& 0& 0\\ -1& -2& -1\end{array}\right]$$

23 $${M}_{xy}=\left[\begin{array}{ccc}0& 2& 2\\ -2& 0& 2\\ -2& -2& 0\end{array}\right]$$

Both ${M}_{x}$ and ${M}_{y}$ operator from Equaiton (15) and Eq. (21) are added to for a composit form of filter. This compiste form generatre “x” and “y” gradient effect as whole along diagonal direction is represented in Eq. (24).

24 $${M}_{xy}=2\left[\begin{array}{ccc}0& 1& 1\\ -1& 0& 1\\ -1& -1& 0\end{array}\right]$$

COFE (or VHT-FE) is designed using a virtual hexagonal trellis overlaid on a predefined square lattice. This feature extractor is particularly effective for regular patterns of the input image. For WBC smear images, these regular patterns are observed, demonstrating the repetition of identical patterns at multiple locations within the image. For such regular patterns, VHT-FE is used to extract gradient features for nucleus and infection analysis, capturing the discriminative characteristics present in the WBC smear image.

The presented feature extractor gathers features in three directions: x, y, and xy diagonal directions at 45- and 135-degree angles. The inclusion of virtual pixels in this proposed feature extractor ensures a low-noise environment and superior accuracy. These virtual pixels help mitigate the blurring effect caused by neighboring pixels and allow for the extraction of more features along the diagonal direction.

The COFE feature extractor yields between 320 and 350 features, depending on the image size and illumination effects. The next step involves labeling and transforming a fusion matrix into a single fused feature vector.

### Feature selection of pretrained model (PTM) WITH ACS

**Figure 8:**
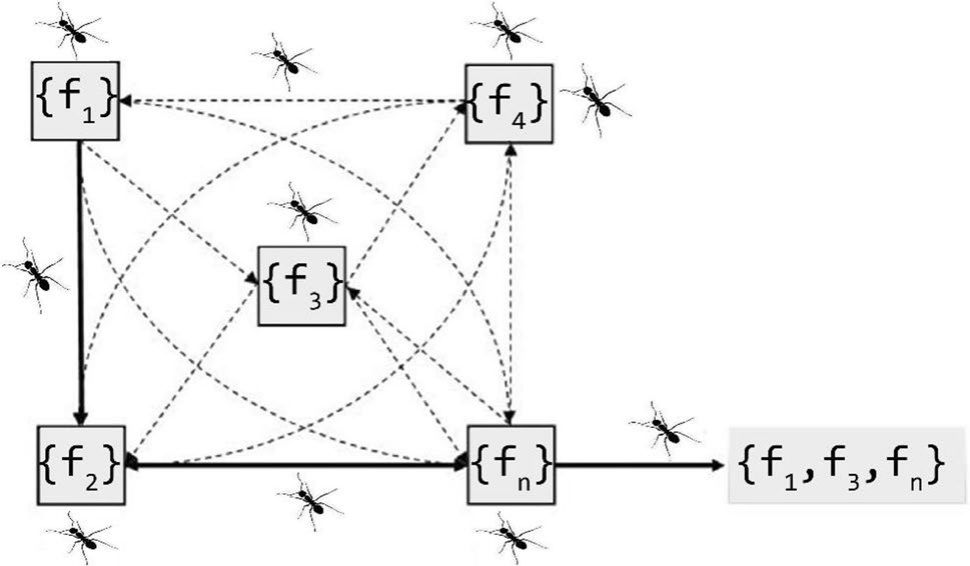
Feature selection using ACS optimizer algorithm.

The Graft Net's fully connected layer produces 4096 optimal features. However, using the complete vector of features at once can increase time complexity. To address this, the ACO technique^54,55^ is applied to the features extracted from the Graft Net, resulting in an average of 1000 optimal features being selected. Within the feature vector subspace, various combinations of subsets are fused together. The fused vector derived from these subspaces is then entered into the classifier, optimizing the classification process. This approach allows for the utilization of a subspace feature vector space. The features gotten from these two subspaces are collected and merged in a sequential manner, generating a fused vector. The process of selecting suitable features using ACO is depicted in Fig. 8.

### Feature fusion (graftnet and VHT-FE)

**Figure 9:**
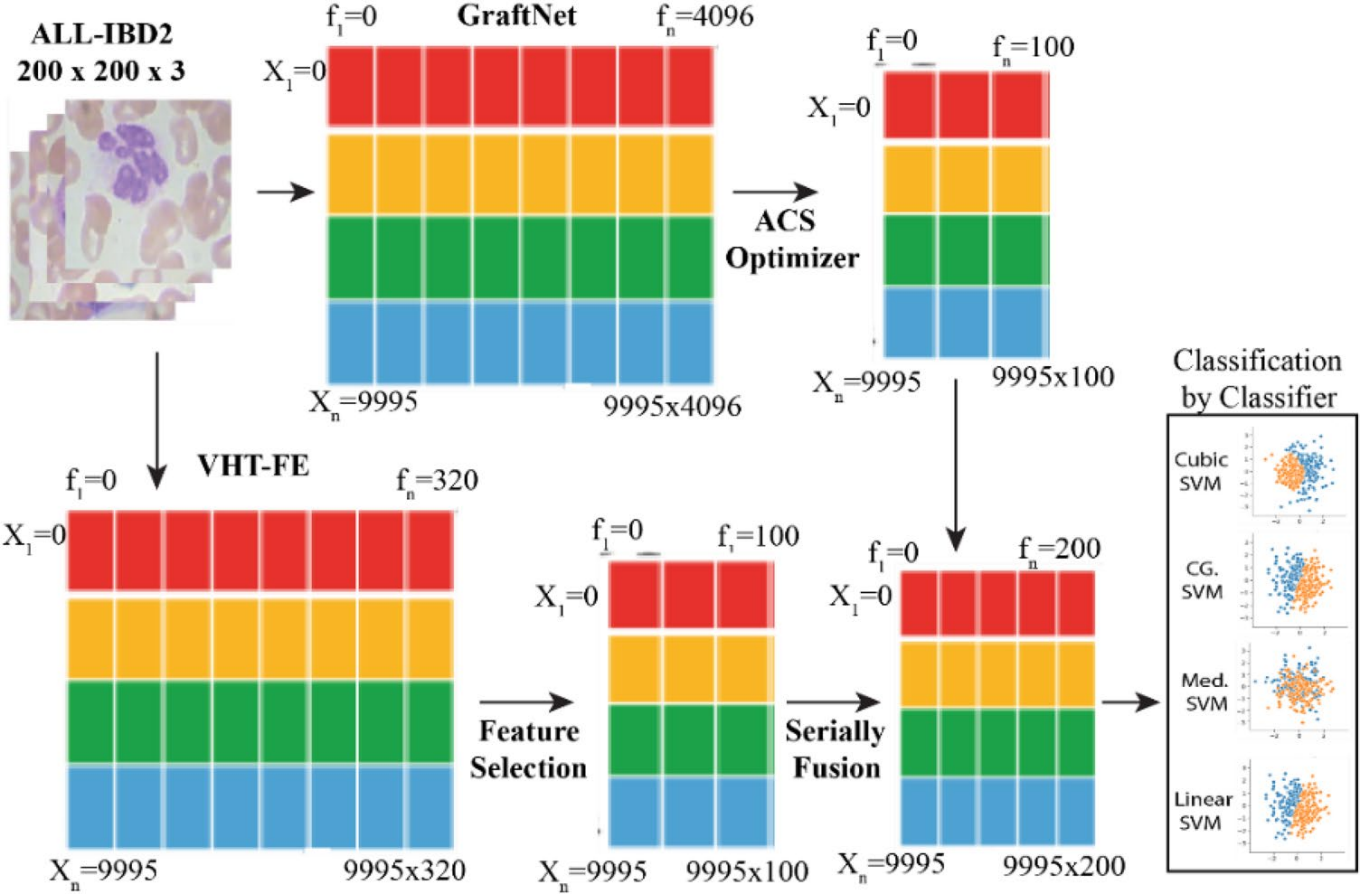
Feature selection and fusion of Graftnet and VHT-FE features.

The process of feature fusion from two models is depicted in Fig. 9. Initially, features are mined from the ALL-IBD2 dataset using the GraftNet model. From the complete vector of features, 100 optimal features are optimized using the ACO technique. These optimal features are then fused with the VHT-FE vector. The fusion of the selected features from both models results in a final fused feature vector consisting of 200 features. This fused vector is subsequently fed into the classifier for the purpose of WBC classification.

### Classification

The mentioned classifiers have been tested for efficiency and classification time using the fused vector from the preceding section. The selected classifiers include various variants of SVM and KNN classifiers.

Each classifier has different variants tailored for time and feature-based analysis. The SVM variants include linear SVM, quadratic SVM, fine Gaussian SVM (FGSVM), medium Gaussian SVM (MGSVM), Coarse Gaussian SVM (Cr. GSVM), and cubic SVM. Similarly, the KNN variants include cosine KNN (CKNN), Coarse KNN (Cr. KNN), and fine KNN (FKNN).

These classifiers have been evaluated based on various performance evaluation parameters. The experimental results indicate that three SVM variants perform better in the classification of WBC. These three variants are cubic SVM, Coarse Gaussian SVM, and Quadratic SVM.

The optimal results achieved using the fused feature range from 750 to 850. Among the three SVM variants, cubic SVM and Coarse Gaussian SVM demonstrate the highest accuracy. The quadratic SVM is observed to be in the third position in terms of performance.

## Results and discussion

The model has been thoroughly tested and validated using SVM, KNN, and tree-based classifiers. The obtained results depend on the selection of feature subsets for the classifier. Optimal results were achieved with a classification accuracy ranging from 98.9 to 99.6%. This accuracy range was observed when selecting feature subsets consisting of 520–1020 features. This section provides detailed information about the dataset and the environment variables used for the performance analysis. All the training, testing, and validation tests were conducted on a machine running Microsoft Windows version 10 with an Intel Core i7 processor, 8 GB RAM, and a Radeon 2G Graphics card. MATLAB 2021a was used as the primary programming tool for the entire duration of working with deep CNN and evaluating the proposed feature extraction method.

### Dataset

**Figure 10:**
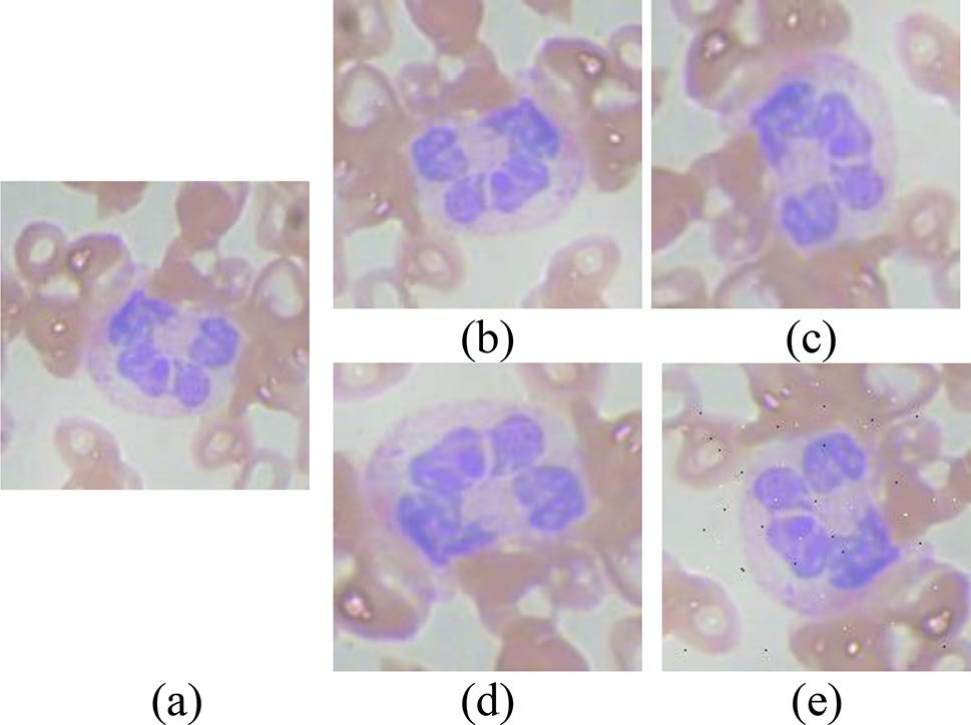
Dataset augmentation: (**a**) Original image (**b**) Rotated right 90 degrees, (**c**) Flipped image right, (**d**) Flipped image down 270 degrees (**e**) Salt and paper noise added image.

The presented dataset of WBC in this work is ALL-IBD2 with sample images shown in Fig. 10. This dataset is publically available for research provided by Kaggle^56^. It contained WBCs four classes: eosinophil, lymphocyte, monocytes, and neutrophil. Each class contains approx. 600 ~ 650 jpg images of size 320 × 240 pixels each shown in Table 1.

**Table 1:** WBCs publically available dataset.

Class	Before Augmentation	Augmented	Training Image	Testing Image
Eosinophil	630	2497	2497	622
Lymphocytes	615	2483	2483	620
Monocytes	540	2478	2478	620
Neutrophils	598	2498	2498	624

The proposed method is a modified version of deep learning methods, which typically require a large dataset for optimal performance. In this study, the selected dataset consists of approximately 2400–2500 images in each class, resulting in a total dataset size of [total number of classes multiplied by 2400–2500]. To enhance the performance of deep learning methods, data augmentation techniques were applied, resulting in an increased number of images to approximately 10,000 in the complete dataset.

Four augmentation techniques are used on sample images shown in Fig. 10a, mirroring vertical shown in Fig. 10b and horizontal in Fig. 10c, flipping horizontally presented in Fig. 10d, and flipping vertically in Fig. 10e.

### Performance evaluation parameters

The performance evaluation includes the analysis of various parameters such as True Positive (TP), False Positive (FP), True Negative (TN), and False Negative (FN), along with metrics like accuracy, sensitivity, specificity, precision, F-Measure (FM), and G-Measure (GM). These parameters are discussed Section 2.3, along with visualizations of the training phase.

### Observation from experiments

Different experiments are conducted for multiple combination of feature vectors as listed in Table 2.

**Table 2:** Summary of experiments performed.

Experiment #	GraftNet Features	VHT Features	Predictor fuse feature vector	Cubic-SVM Accuracy %	C. Gaussian -SVM Accuracy %
1	100	100	200	99.99	99.92
2	200	200	400	99.98	99.76
3	250	300	500	99.97	98.99
4	300	320	600	99.98	99.00
5	400	320	720	99.84	97.83
6	500	320	820	99.62	97.36
7	600	320	920	99.33	96.52
8	750	320	1070	98.90	95.72

The analysis of fused features was directed to assess the potential outcomes. The manuscript presents a summary of selected major experiments. In this section, the first eight outcomes are listed. The highest accuracy percentage was achieved by utilizing a fused feature vector consisting of 200–600 features across all eight experiments.

In the first experiment, 100 features were selected from both the Graft Net model and VHT-FE. These selected features were compound to create a fused feature vector, which was then castoff for ordering. The obtained classification accuracy was 99.99% using cubic SVM and 99.92% using Coarse Gaussian SVM (CG-SVM), as shown in Table 3, experiment 1.

**Table 3:** Summary of experiments performed.

Exp #	Graft Net	VHT- FE	Fused Features	Classifiers	Acc %	Sen %	Sp %	PPV %	NPV %	Error %
1	100	100	200	Cubic SVM	99.99	99.98	99.99	99.97	99.98	0.001
				CG. SVM	99.92	99.95	99.97	99.99	99.98	0.001
				Med. SVM	99.87	99.72	100	100	99.91	0.0013
2	200	200	400	Cubic SVM	99.98	99.99	99.98	99.97	100	0.01
				CG. SVM	99.72	99.76	100	100	99.92	0.037
				Med. SVM	98.38	98.36	99.93	99.80	99.45	0.162
3	250	250	500	Cubic SVM	99.98	99.99	99.99	99.99	99.99	0.01
				CG. SVM	98.99	98.96	99.97	99.92	99.65	0.0101
				Med. SVM	96.75	96.76	99.81	99.42	98.92	0.0325
4	300	300	600	Cubic SVM	99.98	100	100	100	100	0.0002
				CG. SVM	99.00	98.68	99.97	99.92	9956	0.0100
				Med. SVM	94.60	93.71	99.12	97.26	97.92	0.0540
5	400	320	720	Cubic SVM	99.82	99.76	100	100	100	0.0016
				CG. SVM	97.83	97.44	99.92	99.75	99.15	0.0227
				Med. SVM	88.68	89.87	95.25	86.37	96.56	0.1132
6	500	320	820	Cubic SVM	99.62	99.72	100	100	99.91	0.0038
				CG. SVM	97.23	97.36	99.84	99.51	99.12	0.0277
				Med. SVM	81.30	88.03	86.53	68.62	95.57	0.187
7	600	320	920	Cubic SVM	99.33	99.48	99.97	99.92	99.83	0.0067
				CG. SVM	96.35	96.52	99.76	99.26	98.84	0.0365
				Med. SVM	70.58	85.58	74.45	52.86	93.91	0.2942
8	750	320	1070	Cubic SVM	98.90	98.68	99.96	99.88	99.56	0.0110
				CG. SVM	95.72	95.27	99.56	98.63	98.44	0.0428
				Med. SVM	59.32	85.82	60.60	42.17	92.74	0.4068

In the next experiment, 200 features were selected from each model, resulting in a fused feature vector of size 400. The classification accuracy using this vector was 99.98% for cubic SVM and 99.72% for Coarse Gaussian SVM, as presented in Table 3, experiment 2.

In experiment 3, 250 features were picked from both the Graft Net and VHT feature extractor. The 250 features from Graft Net were optimized using ACS and then merged to form 500 features. This attained a classification accuracy of 99.98% using cubic SVM and 98.99% using Coarse Gaussian SVM, as registered in Table 3, experiment 3.

300 ACS-optimized features were collected from GraftNet, while 300 features were collected from the proposed VHT feature extractor. These features were fused to create a feature vector of size 600, which was then used for classification. The classification accuracy of this fused feature vector using different variants of SVM is presented in Table 3, experiment 4. The cubic SVM classifier achieved a classification accuracy of 99.98%, while the Coarse Gaussian SVM achieved an accuracy of 99.00%.

Throughout the series of experiments, minor changes in accuracy were observed. These changes can be attributed to the small number of features added to the fused feature vector. In the preceding experiments, features were fused in an equal ratio from both architectures. However, in the VHT feature extractor, only 320 features were collected. In the upcoming experiments, the optimized features from GraftNet were incrementally increased by 100 features, while the available features from the VHT architecture (320 features) were fused. In this specific experiment (experiment 5), 400 features were collected from GraftNet and the available 320 features were used to form a vector of size 720. This vector was afterwards used for classification, and Table 3 shows the achieved accuracy of 99.82% using cubic SVM and 97.83% using Coarse Gaussian SVM.

In the experiments conducted so far, fused feature vectors ranging from 200 to 600 features were obtained by iteratively selecting 100 features from both the GraftNet and VHT architectures using the ACS algorithm. Currently, there are 320 features available from the VHT feature extractor and 4096 features from GraftNet. The fused feature vector is incremented by an additional 100 features from the previous GraftNet feature vector (totaling 500 features), and the 320 available features from VHT-FE are serially merged. This results in a fused feature vector of size 820, which is then used for performance evaluation by the classifier. The results presented in Table 3, experiment 6, show that cubic SVM outperforms other classifiers with an accuracy of 99.62%, while Coarse Gaussian SVM achieves an accuracy of 97.23%. It is observed that on increase of features, the accuracy gradually decreases after reaching a certain stage of consistent accuracy.

Similarly, in the experiment performed using 920 fused features, it is observed that the classification accuracy gradually decreases. In this experiment, 600 features from GraftNet and the available 320 features from VHT-FE are serially merged to create a vector of size 920, which is further used for evaluation by the classification learner. The variants of SVM perform well with higher accuracy compared to other classifiers. Specifically, cubic SVM achieves an accuracy of 99.33%, and Coarse Gaussian SVM achieves an accuracy of 96.52%. These two best accuracies are listed in Table 3, experiment 7.

The true positive values (TPV) for the four classes are highlighted on the diagonal of the confusion matrix. Overall, every class exhibits a very good recognition rate. The class "monocytes" has the highest accuracy, while "neutrophils" and "eosinophils" have slightly lower accuracies. This observation is also supported by the area under the curve (AUC) and the receiver operating characteristic (ROC). The AUC is analyzed and observed to be nearly 99% for the two best observations, which are represented in the confusion matrix in Tables 4 and 5

**Table 4:** Confusion matrix generated over 720 fused features on all-IBD2 dataset.

**True Classes**				
EOSINOPHILS	**2496**	1	0	0
LYMPHOCYTES	0	**2483**	0	0
MONOCYTES	0	0	**2478**	0
NEUTROPHILS	0	0	1	**2498**
	EOSINOPHILS	LYMPHOCYTES	MONOCYTE	NEUTROPHIL
	**Predicted Classes**

**Table 5:** Confusion matrix generated over 820 fused features on all-IBD2 dataset.

**True Classes**				
EOSINOPHILS	**2497**	0	0	0
LYMPHOCYTES	0	**2482**	1	0
MONOCYTES	0	0	**2478**	0
NEUTROPHILS	0	0	0	**2499**
	EOSINOPHILS	LYMPHOCYTES	MONOCYTE	NEUTROPHIL
	**Predicted Classes**			

The final experiment aimed to evaluate the fused features from both the GraftNet and VHT-FE architectures. In this experiment, a total of 1070 features were serially fused to create a fused vector. The GraftNet architecture contributed 750 features, while the VHT-FE architecture contributed 320 features. Although the accuracies observed in this experiment were slightly lower compared to the previous experiments, they still remained at a high level of performance. The variant of SVM classifier achieved accuracies of 98.90% and 95.72% for cubic SVM and Coarse Gaussian SVM, respectively, as shown in Table 3, experiment 8. The confusion matrix, which represents the AUC and ROC of the data, is also presented in Table 5.

**Figure 11:**
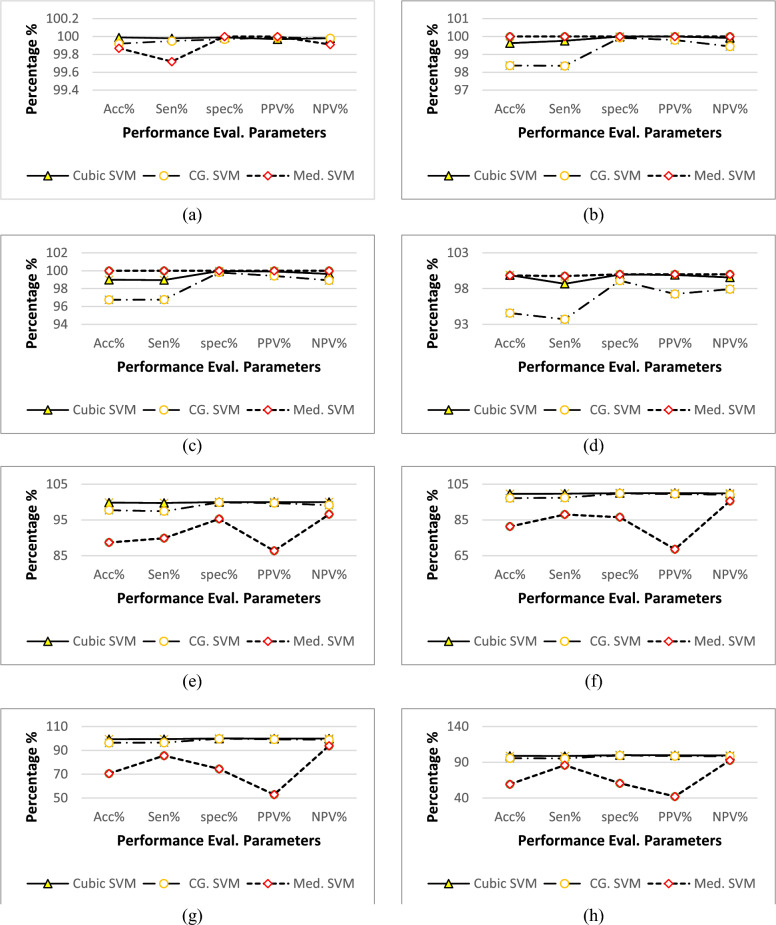
Performance evaluation parameter analysis over different sub-space fused feature vectors (FFV) (**a**) 200 FFV (**b**) 400 FFV (**c**) 500 FFV (**d**) 600 FFV (**e**) 720 FFV (**f**) 820 FFV (**g**) 920 FFV (**h**)1070 FFV.

Despite having only 320 extracted features from VHT-FE, the fusion with GraftNet features yields optimal performance. However, the analysis of fused features in Table 3, experiments from 1 to 8, indicates that adding more features from GraftNet leads to a drop in overall accuracy. The complete results and analysis, including the parametric values and accuracy of predictors at each iteration of different feature subsets, are presented in Fig. 11.

**Figure 12:**
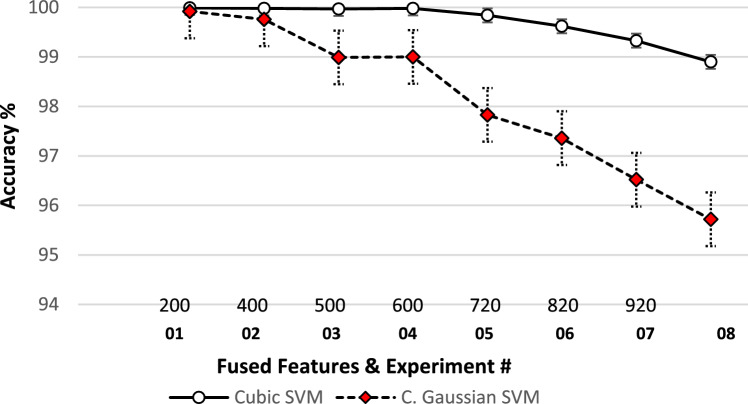
Fused features vectors vs accuracy.

The selected features are serial merged to form a vector listed in fused features column in Tables 3. All results are extracted using experiments series and discussed in preceding section "observation from experiments". The summary of observations in Fig. 12 clearly shows the performance results of fusing 500 + 320 features to form an 820-feature matrix. It is observed that the fused features matrix (FFM) of 720 features and 820 features achieve approximately the same accuracy percentage with very small variations.

**Figure 13:**
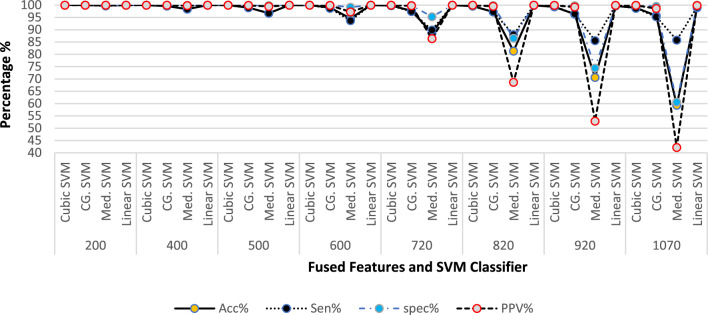
Performance percentage vs features vectors and multiple SVM classifiers.

In the proposed work, we witnessed that every model has an effective threshold beyond which its performance starts to decline. High accuracy is attained by using the fused feature subsets ranging from 720 to 820. This range has revealed optimum performance in experiments. On addition of a large number of features, it leads to a loss of prediction capability due to high variance. On the other hand, if the model is trained with limited features, it results in a large bias. Both scenarios negatively affect the performance of the prediction. To attain high prediction accuracy, it is important to deploy effective feature extraction techniques in the training model. This allows us to have a balance between bias and variance to get the maximize accuracy. The comparative analysis of classifiers based on the fused features and their accuracy is presented in Fig. 13.

As the number of features increases, the accuracy, sensitivity, specificity, and positive predicted values (PPV) decrease. However, it is worth noting that even with a decrease in accuracy, the performance evaluation parameters remain at high levels. For instance, over 200 fused features, all variants of SVM perform very well with accuracies of 99.99%. From 200 to 600 fused features, the performance evaluation results remain consistently high, ranging from approximately 99.98–99.99% accuracy. However, as the number of features increases beyond 720 fused features from GraftNet, there is a gradual decrease in accuracy. This trend continues up to 1070 fused features.

**Figure 14:**
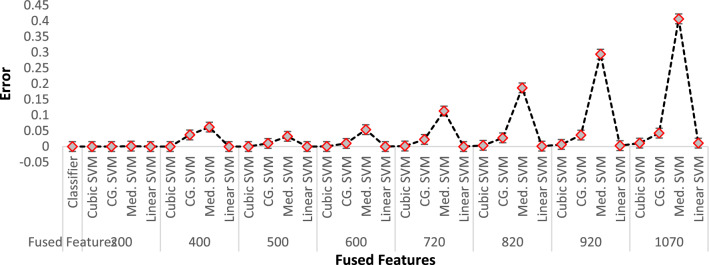
Classification error of different SVM variants.

During the series of experiments, the classification error of numerous *SVM* variants was also observed. It was noted that as the fused features upsurges, the classification error of these classifiers also rises. This trend is consistent across all variants of SVM. Although each variant of SVM classifier may exhibit a different error ratio, the general observation is that the error tends to increase with an increase in fused features. The finding is depicted in Fig. 14.

### Discussion and suggestions

The main focus of the manuscript is to analyze WBC images from the ALL-IBD2 dataset using a machine learning approach. In order to improve the performance of infection detection in image analysis, two different techniques, namely Graft Net and the proposed architecture virtual hexagonal trellis, are used in combination for experimentation. The Graft Net model is pre-trained using the CIFAR dataset, which consists of 100 classes. The features of the WBC images are extracted from Graft Net and then passed through the ACO algorithm for feature optimization.

In the proposed method, a virtual hexagonal trellis-based operator is modeled and convolved over the same WBC images as in the Graft Net process. The features extracted from the virtual hexagonal trellis-based operator are already of a limited number. These features are directly fused with the optimized features from Graft Net to form a fused feature vector. This fused feature vector is then fed to variants of SVM and KNN classifiers for analysis.

**Figure 15:**
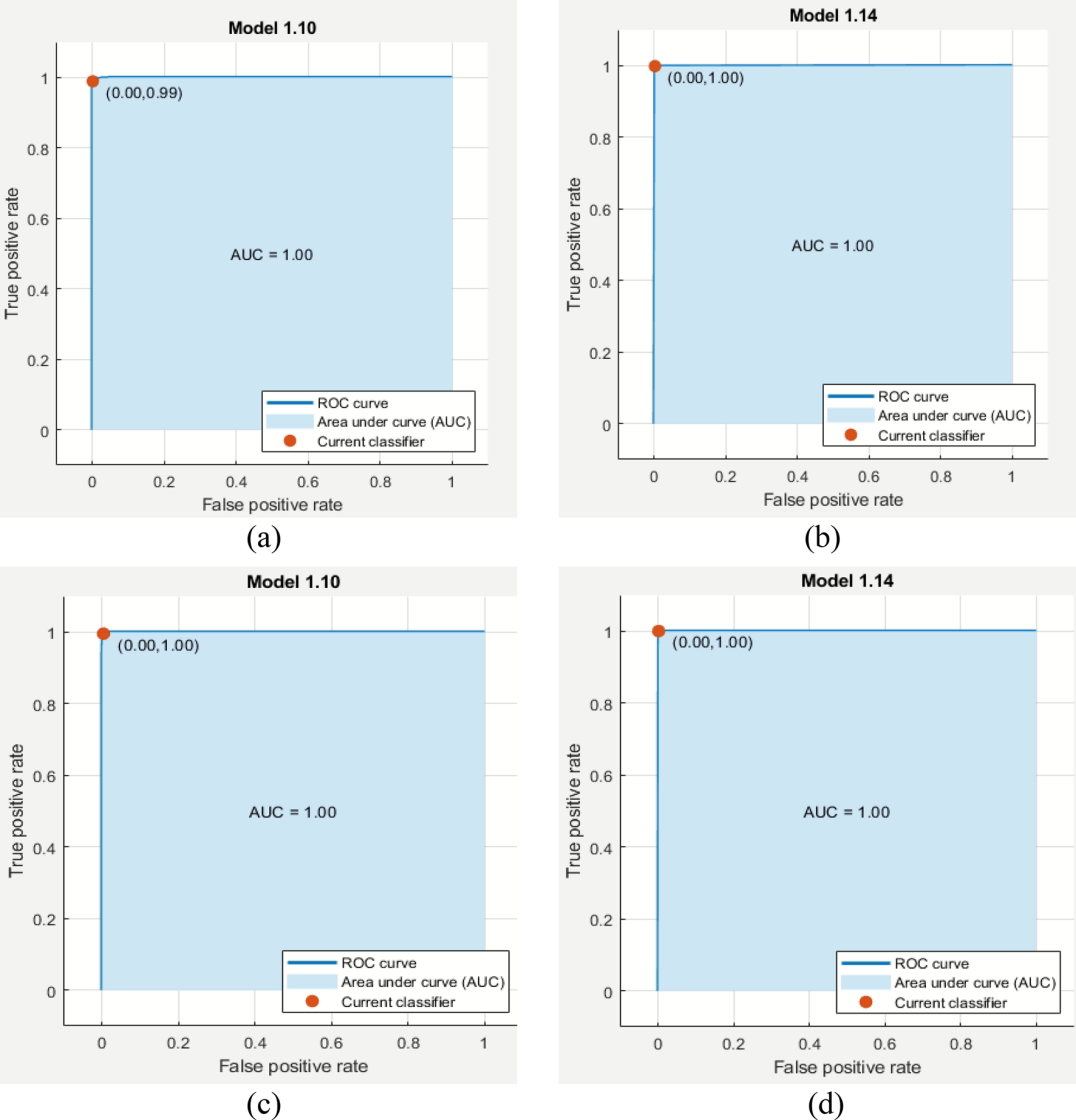
ROCs and AUCs of all classes of the best outcome of multiple SVM classifiers.

The optimal accuracy is achieved for the WBC class, specifically for monocytes, compared to other classes such as neutrophils and eosinophils. This observation is also supported by the area under the curve (AUC) and the receiver operating characteristic (ROC) curve shown in Fig. 15. The AUC is analyzed and observed to be nearly 99%, indicating a high level of performance.

The process of feature fusion has been described in the previous section and summarized in Table 2. The outcomes presented in Table 3 demonstrate the effectiveness of the projected model in terms of accuracy (Ac), sensitivity (Si), precision (Pr), specificity (Sp), F1 measures (FM), and G-mean (GM). The observations from the experiments indicate that as the fused features rises, the presentation of the classifier also improves. However, there is a threshold beyond which further increasing the number of fused features negatively impacts the classifier's performance. The composite model with 720 and 820 fused features is found to perform better overall.

The tree-based classifier showed poor performance on the WBC dataset, achieving less than 45% accuracy overall. The accuracy of KNN variations, ranging from 40 to 65%, is not discussed in this manuscript. The SVM-based classifier, on the other hand, achieved the highest accuracy, up to 99.99% depending on the duration and speed of training. It is observed and inferred that SVM variations perform better than KNN and tree-based classifiers and have significantly shorter prediction times.

The outcomes show that the model with proposed virtual hexagonal trellis based feature extractor (VHT-FE), attained a better performance.

## Conclusion

The proposed work presented the devised virtual hexagonal lattice (VHT) structure and deep GraftNet architecture for WBC prediction. GraftNet is a pre-trained CNN model trained on the third party CIFAR dataset. The WBC dataset is castoff to acquire features using the GraftNet. Data augmentation in applied to improve the learning mechanism. The ACO algorithm is employed to acquire the best features from the. These features are serially fused and the fused vector is then fed to various variants of the SVM classifier for prediction. Multiple experiments are conducted using different feature subsets to evaluate the anticipated model. The results indicate that when using a fused feature vector of 720/820 features, the classification accuracy reaches 99.91% and 99.99%, respectively. In terms of performance accuracy, there is a notable distinction between SVM and KNN classifiers. The suggested composite model demonstrates superior performance accuracy in the domain of medical image classification. The model is optimized through various techniques, including data augmentation, feature extraction, and feature selection, to achieve higher accuracy compared to other variations of the dataset.

Future research in this field could incorporate advanced methodologies such as quantum learning and manifold learning techniques to further enhance the performance and insights of the proposed model.
